# Does in-transit feeding of day-old chicks enhance potato peel meal utilization? Impact on transport stress, gastrointestinal organ weight and performance in broilers

**DOI:** 10.1016/j.psj.2026.106809

**Published:** 2026-03-17

**Authors:** Xujie Li, Jing Lu, Bruce Rathgeber, Anna Rogiewicz, Stephanie Collins

**Affiliations:** a Department of Animal Science and Aquaculture, Faculty of Agriculture, Dalhousie University, Truro, NS B2N 5E3, Canada; b Department of Animal Science, University of Manitoba, Winnipeg, MB R3T 2N2, Canada

**Keywords:** Potato peels, Enzyme supplementation, Early feeding, Productivity, Corticosterone, Broiler

## Abstract

The use of waste-derived feed ingredients to support sustainable poultry production has gained increasing attention. The potential of incorporating potato peels, an agro-industrial by-product, into broiler feed remains unexplored. Study 1 (preliminary study) evaluated the effects of dietary inclusion (0% vs. 10%) of potato peel meal (**PP)** with or without a multi-carbohydrase (**E1**) supplementation on growth performance of Cobb 500 broilers from d 14 to 33 of age in a 2 × 2 factorial arrangement (n=6). Incorporating 10% PP into diets suppressed growth and increased gizzard weight (*P* ≤ 0.05) compared to those fed 0% PP. Building on these findings, Study 2 was conducted to investigate whether the negative effects on growth could be mitigated by reducing PP inclusion level, supplementing a modified multi-carbohydrase (**E2**) and providing nutrients during transportation. A total of 624 newly-hatched Ross 308 chicks were assigned to a 2 × 4 factorial design (n=3) with two in-transit feeding practices (**AFW**: access to feed and water during transportation vs. **NFW**: no access to feed and water) and four dietary treatments including: 0% PP (control), 5% PP, 5% PP+E2, and 10% PP+E2, which were provided *ad libitum* from d 1 to 33. AFW improved chick quality (*P* ≤ 0.05) with an increased body weight gain, higher cloaca temperature, and lower plasma corticosterone level post-transportation. In addition, AFW increased body weight and reduced mortality by d 8 of age (*P* ≤ 0.05). Regardless of in-transit feeding, broilers fed 10% PP+E2 or 5% PP exhibited comparable body weight, body weight gain, feed intake and FCR to those fed control diet. Among AFW birds, 5% PP+E2 improved FCR (*P* ≤ 0.05) compared with 5% PP and 10% PP+E2 during d 26-33 of age. These findings suggest that PP can be included up to 10% when supplemented with E2 without compromising growth performance. Furthermore, the in-transit feeding practice alleviates transportation stress, supports early growth and enhances FCR in birds fed moderate levels of PP with enzyme supplementation.

## Introduction

The rising cost of traditional feed ingredients, such as corn and soybean, coupled with fluctuations in their availability, presents a significant challenge to sustainable poultry production. In this context, alternative feed ingredients derived from agro-industrial by-products or waste streams, especially those available locally, have several advantages. Their inclusion in poultry diets has strong potential to reduce feed cost (Alqaisi et al., 2017), which typically accounts for 50-80% of total production expenses (Schnepf, 2011). Additionally, this approach supports sustainable poultry production by transforming waste into valuable nutrients, thereby reducing waste disposal pressure and its associated environmental impacts (Alqaisi et al., 2014; Leinonen and Kyriazakis, 2016).

Potato peels are one of the underutilized by-products that offer great promise as an alternative feed ingredient in poultry diets. In 2023, total potato production in North America is approximately 265 million tonnes (FAOSTAT, 2025), with up to 10% lost (fresh weight basis) as peel waste during industrial processing (Mäder et al., 2009). These wasted potato peels can be a valuable source of nutrients, although their composition varies based on the potato variety, peeling method, and subsequent processing. Potato peels are high in dietary fiber (30-50% total fiber, mostly insoluble), and the non-starch polysaccharide (**NSP**) fraction mainly consists of cellulose, hemicellulose and xylose (Camire et al., 1997). These insoluble NSP play a beneficial role in poultry diets, as moderate amounts improve digestion and absorption by stimulating gizzard development, which in turn enhances the mechanical breakdown of feed, promotes mixing with digestive enzymes, and slows the passage rate of digesta through the digestive system. In addition to fiber, potato peels contain approximately 8-18% crude protein and are a significant source of potassium, zinc, iron and phenolic compounds (Elkahoui et al., 2018; Nelson, 2010; Vaitkevičienė et al., 2019). Phenolic compounds are particularly concentrated in the peels, with levels up to ten times higher than those found in whole potatoes (Albishi et al., 2013). Repurposing potato peels as a poultry feed ingredient could reduce the environmental impacts of waste management and provide a cost-effective feed ingredient option for poultry producers.

Despite the nutritional potential of potato peels, limited studies have investigated their use in poultry diets. The high dietary fiber, particularly the NSP fraction, may reduce the availability of nutrients for digestion and utilization by chickens. Several studies have demonstrated that exogenous carbohydrase supplementation can degrade NSP in cereal-based diets and improve the digestibility of starch and NSP in the small intestine of broilers (Kiarie et al., 2014; Meng et al., 2004; Munyaka et al., 2016). Exogenous carbohydrase supplementation, in combination with protease, can hydrolyze specific NSP fractions, reduce digesta viscosity and disrupt protein-carbohydrate complexes, thereby mitigating the antinutritional effects of fibrous ingredients and improving nutrient accessibility and utilization (Aftab and Bedford, 2018). However, considerable evidence shows that effective NSP degradation depends on the types and activity of the enzymes, as different fiber sources require a specific enzyme profile (Meng and Slominski, 2005; Niu et al., 2022; Slominski, 2011). To our knowledge, a multi-carbohydrase supplement tailored to potato peels has not been identified in the literature, but such an enzyme blend is essential to allow their use as a feed ingredient in poultry diets.

The early life represents a critical window to support gut development and early nutrition strategies can have lasting impacts on the health and growth of chickens in later stages of bird’s life span (Rubio, 2019). At hatch, the digestive system of chicks is still immature with limited digestive enzyme secretion (Nitsan et al., 1991), which limits their ability to digest fibrous ingredients like potato peels. Previous studies have demonstrated the positive impacts of early feeding practice, including *in ovo* nutrient administration (administering nutrients into developing embryos before hatch) and in-hatcher feeding (providing feed immediately after hatch), on enhancing chick quality and stimulating the secretion of digestive enzymes, growth factors, and neuronal signals that promote gastrointestinal development (Boussaada et al., 2020; Liu et al., 2020; Uni et al., 2003). Additionally, hatchlings may experience feed and water deprivation for up to 72 h before having access to a protein- and carbohydrate-based feed (Willemsen et al., 2010). Delayed access to feed for up to 48 h post-hatch, which commonly occurs during hatchery processing and transportation, has been associated with delayed gut development and poor production performance (Geyra et al., 2001; Li et al., 2022). While most early feeding studies focused on providing nutrients via *in ovo* administration or examining the effects of delayed feeding on growth performance, limited research has explored the role of nutritional management during the transit period between hatcheries and broiler chicken barns on stress response and gastrointestinal organ development. In addition to nutrient deprivation, transportation exposes day-old chicks to multiple stressors, including space restriction, suboptimal truck microclimate conditions, prolonged transport duration and vibration. These factors can activate physiological stress response, negatively affect welfare, and impair subsequent performance (Mitchell and Kettlewell, 2009). It remains pivotal to investigate whether the in-transit feeding practice can mitigate transport-related stress, accelerate gut development and improve chicks’ abilities to utilize high-fiber feed ingredients.

We hypothesized that in-transit feeding practice (access to feed and water during transportation) could mitigate transport stress, promote gastrointestinal tract development and improve early growth performance. Furthermore, in-transit feeding practice may improve utilization of potato peel meal (**PP**) as an alternative carbohydrate source when supplemented with a suitable multi-carbohydrase without compromising growth performance. The objectives of this study were 1) to examine the effects of nutrient access during transportation on chick quality, stress response, gastrointestinal organ development and growth performance in broiler chickens, and 2) to evaluate the potential of incorporating PP into broiler diets, focusing on the effects of in-transit feeding and varying levels of PP supplemented with multi-carbohydrase on gastrointestinal organ weight and production parameters.

## Materials and methods

The present animal experiment was approved by the Animal Care and Use Committee of Dalhousie University, Faculty of Agriculture (File#: 2024-061), and all protocols were performed according to the guidelines of the Canadian Council on Animal Care (CCAC, 2009).

### Study 1: preliminary evaluation of potato peel meal in grower and finisher phases

Fresh potato peels were obtained from McCain Foods Canada (Florenceville-Bristol, NB, Canada) and oven-dried at 65°C for 24 h, then ground using a hammer mill equipped with 4 mm screens. The nutrient content and NSP profile of the PP are presented in Table 1. PP was ground using a coffee grinder and analyzed for dry matter (method no. 935.29), crude protein (method no. 990.03) and mineral composition (method no. 968.08) according to AOAC standard procedures (AOAC, 2005). Crude fat was determined using Ankom XT extraction (AOAC, 2005), and neutral detergent fiber (NDF) and acid detergent fiber (ADF) were determined using Ankom Fibre Analyzer (AOAC, 2005). The NSP components were determined by gas-liquid chromatography (component sugars) using SP-2340 column and Varian CP-3380 GC (Agilent Technology, Mississauga, ON, Canada) and by colorimetry (uronic acids) using a Biochrom Ultrospec 50 (Biochrom Ltd., Cambridge, UK) according to the procedure described by Englyst and Cummings (1988) with some modifications (Slominski and Campbell, 1990). A total of 576 one-day-old Cobb 500 mixed-sex broiler chicks were obtained from a commercial hatchery. Upon arrival at the poultry barn (Atlantic Poultry Research Institute, Dalhousie University, Faculty of Agriculture, Truro, NS, Canada), the chicks were group weighed and placed into 24 floor pens (2.19 m x 1.00 m) with new wood shavings at a depth of 4 cm in a single broiler housing unit. Each pen housing 24 birds served as an experimental unit. From d 0 to 14, all chicks were provided with a nutritionally balanced starter diet in crumble form (AME: 2950 Kcal/kg, crude protein: 23%). At 14 d of age, birds with similar body weight were randomly assigned to one of four dietary treatments in pellet form: 1) the basal diet (**0PP**), 2) 0PP supplemented with multi-carbohydrase (**E1, 0PP+E1**), 3) PP replacing 10% of the corn meal in the basal diet (**10PP**), and 4) 10PP supplemented with E1 (**10PP+E1**) (Table 2). The E1, Superzyme-CS 2x, was provided by CBS Bio Platforms Inc. (Calgary, AB, Canada) and added to the respective diets (diets 2 & 4) before diet mixing, at the level of 250 g/t according to the manufacturer’s recommendation. As stated by the manufacturer, one unit of amylase activity (**FAA**) was defined as the amount of enzyme required to break down 5.26 mg of starch per h at 40°C and pH 5.0; one unit of protease activity (**HUT**) was defined as the amount of enzyme that, in one minute at 40°C and pH 4.7, produces a hydrolysate whose absorbance at 275 nm is equivalent to that of a solution containing 0.275 µg/mL tyrosine in 0.006 N HCl; one unit of pectinase activity (**PEC**) was defined as the amount of endo-polygalacturonase that produces 1 µmole of D-galacturonic acid per minute at 40 °C and pH 4.5; one unit of xylanase activity (**XYL**) was defined as the amount of enzyme produces 1 µmole of xylose per minute at 40 °C and pH 4.5; one unit of cellulase activity (**CMC**) was defined as the amount of enzyme produces 1 mg of glucose per hour at 37 °C and pH 4.6; one unit of invertase activity (**INV**) was defined as the amount of enzyme produces 1 µmole of invert sugar per minute at 30 °C and pH 4.7; one unit of glucanase activity (**GLU**) was defined as the amount of enzyme produces 1 mg of maltose per minute at 50 °C and pH 5.0. Each gram of the enzyme product contained: 25,002 FAA units of amylase, 12,897 HUT units of protease, 6,151 PEC units of pectinase, 2,868 XYL units of xylanase, 1,654 CMC units of cellulase, 750 INV units of invertase and 363 GLU units of glucanase. The diets were formulated to meet or exceed the National Research Council (1994) nutrient requirements. Feed and water were provided *ad libitum* throughout the study.

**Table 1:** Analyzed nutrient content and NSP profile of the oven-dried potato peel meal.

Item	Oven dried potato peel meal	
	As-fed basis	Dry matter basis
*Nutrient content*		
Dry matter, %	92.01	-
Crude protein, %	13.50	14.67
Crude fat, %	2.78	3.02
1Metabolizable energy, Kcal/kg	2690	2973
ADF, %	55.18	59.97
NDF, %	67.89	73.79
Ash, %	6.78	7.37
Calcium, %	0.19	0.20
Potassium, %	2.64	2.87
Magnesium, %	0.16	0.17
Phosphorus, %	0.09	0.10
Sodium, %	0.02	0.03
*NSP component*		
Arabinose, %	2.91	3.16
Xylose, %	2.49	2.71
Mannose, %	1.06	1.15
Galactose, %	1.53	1.66
Glucose, %	15.25	16.57
Uronic acids, %	6.55	7.12
Total NSP, %	29.78	32.27

1 ME value of potato peels was calculated as 37.00 x % CP + 81.00 x % EE + 35.50 x % NFE (Abdel-Hafeez et al., 2018)

**Table 2:** Ingredients and nutrient composition of experimental grower and finisher diets (as fed basis) in Study 1 (preliminary study).

Item		Dietary Treatment^4^						
	Grower (15-24 d)				Finisher (25-33 d)			
	0PP	0PP +E1	10PP	10PP +E1	0PP	0PP +E1	10PP	10PP +E1
*Ingredients, %*								
Potato peel meal (PP)^1^	0.00	0.00	10.00	10.00	0.00	0.00	10.00	10.00
Corn	42.19	42.14	33.21	33.16	48.21	48.16	38.82	38.77
Soybean meal	40.71	40.72	38.97	38.98	34.51	34.52	32.85	32.86
Wheat	10.00	10.00	10.00	10.00	10.00	10.00	10.00	10.00
Soybean oil	2.14	2.16	3.04	3.05	2.28	2.30	3.31	3.32
Limestone	1.70	1.70	1.50	1.50	1.70	1.70	1.70	1.70
Dicalcium phosphate	1.50	1.50	1.50	1.50	1.50	1.50	1.50	1.50
Vitamin and mineral mix^2^	0.50	0.50	0.50	0.50	0.50	0.50	0.50	0.50
NaCl	0.50	0.50	0.50	0.50	0.50	0.50	0.50	0.50
Pellet binding agent	0.50	0.50	0.50	0.50	0.50	0.50	0.50	0.50
L-threonine	0.10	0.10	0.10	0.10	0.10	0.10	0.10	0.10
L-lysine	0.02	0.02	0.04	0.04	0.07	0.07	0.09	0.09
DL-methionine	0.13	0.13	0.14	0.14	0.13	0.13	0.14	0.14
Superzyme-CS 2x (E1)^3^	0.00	0.025	0.00	0.025	0.00	0.025	0.00	0.025
Total	100.00	100.00	100.00	100.00	100.00	100.00	100.00	100.00
*Calculated composition*								
Crude protein, %	21.00	21.00	21.00	21.00	19.00	19.00	19.00	19.00
Metabolizable energy, Kcal/kg	3000.00	3000.00	3000.00	3000.00	3050.00	3050.00	3050.00	3050.00
Crude fat, %	4.44	4.46	5.12	5.14	4.65	4.67	5.45	5.47
Lysine, %	1.16	1.16	1.16	1.16	1.06	1.06	1.06	1.06
Methionine, %	0.47	0.47	0.47	0.47	0.44	0.44	0.44	0.44
Threonine, %	0.83	0.83	0.81	0.81	0.75	0.75	0.73	0.73
Calcium, %	0.96	0.96	0.91	0.91	0.94	0.94	0.96	0.96
Phosphorus, %	0.71	0.70	0.68	0.68	0.69	0.69	0.66	0.66
Sodium, %	0.20	0.20	0.21	0.21	0.20	0.20	0.20	0.20

1 Potato peel meal was prepared by drying raw potato peels at 65°C for 24 h, then grinding them to pass through a 4.0 mm screen before being incorporated into the diet. The dried potato peel meal (*as-fed*) used contained 7.99% moisture, 13.50% CP, 55.18% ADF, 67.89% NDF, 2.78% CF, 6.78% Ash, 0.19% Ca, 2.64% Na, 0.16% Mg, 0.09% P, 0.02% Na, 11.58 ppm Cu, 240.48 ppm Mn, and 34.14 ppm Zn.

2 Supplied per kg of diet: 9,750 IU vitamin A, 2,000 IU vitamin D3, 50 IU vitamin E, 3.2 mg vitamin K, 8.6 mg riboflavin, 13.5 mg DL Ca-pantothenate, 0.023 mg vitamin B12, 40 mg niacin, 1.9 mg folic acid, 801 mg choline chloride, 0.25 mg biotin, 5.4 mg pyridoxine, 4 mg thiamine, 120 mg Manganous oxide, 110 mg Zinc oxide, 16 mg Copper sulfate.

3 Each gram of the enzyme product (Superzyme-CS 2x) provided: 25,002 FFA units of amylase, 12,897 HUT units of protease, 6,151 PEC units of pectinase, 2,868 XYL units of xylanase, 1,654 CMC units of cellulase, 750 INV units of invertase and 363 GLU units of glucanase. Each kg of feed contains 6,250 FFA units of amylase, 3,224 HUT units of protease, 1,537 PEC units of pectinase, 717 XYL units of xylanase, 413 CMC units of cellulase, 187 INV units of invertase and 90 GLU units of glucanase.

4 Dietary Treatment: 0PP, basal diet; 0PP+E1, basal diet supplemented with E1; 10PP: PP replacing 10% of the corn meal in the basal diet; 10PP+E1: 10PP diet supplemented with E1.

Birds in each pen were weighed on d 0, 14, 24 and 33. The feed remaining in the feeders was weighed on each weigh day and as mortality occurred. Growth performance was evaluated based on feed consumption, body weight, body weight gain and feed conversion ratio (**FCR**). On d 33 of age, one male bird per pen was randomly selected and euthanized by cervical dislocation to evaluate the development of the gastrointestinal tract. Only male birds were sampled to avoid potential sex-related differences in the measurements. The weight of the crop, gizzard, duodenum, jejunum, ileum, cecum, large intestine and liver was expressed as g per 100 g of body weight.

### Study 2: in-transit feeding and potato peel meal inclusion

**Fig. 1:**
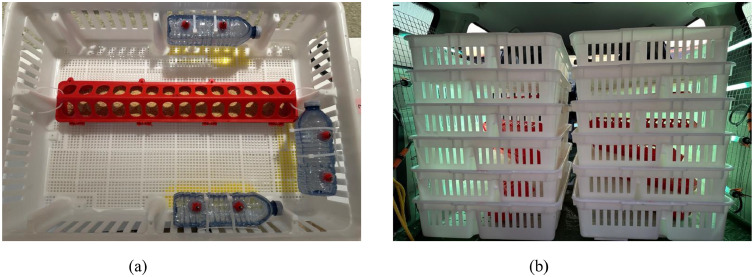
*(a)* Experimental setup showing the placement of feeder and water drinkers within the transportation basket. *(b)* Layout of transportation baskets arranged inside the transportation van during chick transport.

***Experimental Design and Transportation Management*.** A total of 624 Ross 308 mixed-sex day-old chicks were obtained from a commercial hatchery and randomly distributed into 12 transportation baskets (62 cm x 48 cm), with 52 chicks per basket. Each transportation basket (Fig. 1a) was equipped with one 50 cm trough feeder (Little Giant, Miller Manufacturing, Eagan, MN) and three 500 mL water drinkers (2 drinking nipples per drinker). Six transportation baskets were allocated to the in-transit feeding group, which chicks had access to 0% PP starter feed and fresh water *ad libitum* (**AFW**). The feeder and water drinkers in the remaining six transportation baskets remained empty during transportation (**NFW**). Cloaca temperature (10 chicks/basket) and body weight were measured before transportation. The transportation baskets were alternately stacked (Fig. 1b), and chicks were transported for 4 h in a farm van under controlled conditions (temperature: 25°C; relative humidity: 33%; light intensity: 100 lux). The transportation consists of 2 h on highway at 110 km/h and 2 h on rural roads at 50 km/h, representing typical travel conditions and stops between farms.

Immediately after transportation, chicks were group weighed and the cloaca temperature of 10 chicks per basket was measured again. Body weight changes during transport were calculated and expressed as a percentage of pre-transport body weight. The weights of the remaining feed and water were measured to calculate the feed and water consumption during transportation. Two randomly selected chicks per basket used for blood and tissue collections were euthanized via cervical dislocation and yolk sacs were removed and weighed. Approximately 1.5 mL of blood from the jugular vein was collected into a sodium heparin-coated tube. Blood glucose level was measured using a blood glucose meter (ACCU-CHEK Guide, Roche, Germany). Blood samples were kept on ice and centrifuged (2,000 x g, 15 min at 4°C) within 1 h of collection. Plasma corticosterone level was determined by using a commercial Corticosterone Enzyme Immunoassay kit (Arbor Assays^TM^ DetectX® Corticosterone ELISA Kit, K014—H1, Ann Arbor, MI, USA) following the manufacturer’s procedure. The chick quality assessment included yolk-free body weight (chick’s body weight subtracts yolk sac weight), crop fill (score 0: empty; score 1: feed only; score 2: water only; score 3: feed and water, full, soft and rounded), and the weight of yolk sac, liver, pancreas, as well as both intact and emptied gizzard, duodenum, jejunum, ileum and cecum. The relative weight of intestinal segments and organs were expressed as g per 100 g of yolk-free body weight.

***Diets and Bird Management.*** The remaining chicks were placed into 24 floor pens bedded with new wood shavings and managed according to the same experimental setup as described in Study 1. All chicks from the same in-transit feeding treatment were divided into 12 pens and randomly assigned to one of four dietary treatments. A modified multi-carbohydrase blend (**E2**), Superzyme-CC 2.5x, was prepared by CBS Bio Platforms Inc. (Calgary, Canada) and used in Study 2 with a recommended dosage of 200 g/t. Each gram of E2 contains 2,875 XYL units of xylanase, 900 GLU units of glucanase, 542 INV units of invertase, 7,750 HUT units of Protease, 4,500 CMC units of cellulase, 18,125 FAA units of amylase and 60 GAL units of galactanase. The dietary treatments were 1) the basal diet (**Control**), PP replacing 5% of the corn meal in the control diet (**5PP**), 5PP diet supplemented with E2 (**5PP+E2**), and PP replacing 10% of the corn meal in the control diet with E2 supplementation (**10PP+E2**). Dietary composition and calculated nutrients of the experimental diets are presented in Table 3. Starter diets (0-15 d) in mash form, grower diets (16-25 d) in pellet form and finisher diets (26-33 d) in pellet form were provided *ad libitum*. Birds received 18 h of light and 6 h of dark through a 33-d period.

**Table 3:** Ingredients and nutrient composition of experimental diets (as fed basis) in Study 2.

	Starter^4^ (d 0-15)				Grower^4^ (d 16-25)				Finisher^4^ (d 26-33)			
Item	Control	5PP	5PP+E2	10PP+E2	Control	5PP	5PP+E2	10PP+E2	Control	5PP	5PP+E2	10PP+E2
*Ingredients, %*												
Potato peel (PP)	0.00	5.00	5.00	10.00	0.00	5.00	5.00	10.00	0.00	5.00	5.00	10.00
Soybean meal	45.07	44.26	44.27	43.46	45.15	40.49	40.45	37.23	47.33	42.71	42.67	38.05
Corn	38.31	33.65	33.61	29.94	38.84	38.03	38.04	35.78	35.95	35.13	35.14	34.32
Wheat	10.00	10.00	10.00	10.00	10.00	10.00	10.00	10.00	10.00	10.00	10.00	10.00
Dicalcium phosphate	2.05	2.17	2.19	2.66	1.49	1.96	1.98	2.45	2.53	2.97	2.98	3.41
Soybean oil	1.70	2.06	2.06	2.06	1.25	1.26	1.26	1.26	1.15	1.13	1.13	1.14
Limestone	1.28	1.26	1.26	1.24	1.24	1.22	1.22	1.20	1.13	1.13	1.13	1.12
DL-methionine	0.68	0.69	0.69	0.71	0.59	0.61	0.61	0.62	0.50	0.52	0.52	0.53
Vitamin and mineral mix^1^	0.50	0.50	0.50	0.50	0.50	0.50	0.50	0.50	0.50	0.50	0.50	0.50
Pellet binding agent	-	-	-	-	0.50	0.50	0.50	0.50	0.50	0.50	0.50	0.50
NaCl	0.40	0.40	0.40	0.40	0.40	0.40	0.40	0.40	0.40	0.40	0.40	0.40
L-lysine	0.00	0.01	0.01	0.01	0.03	0.03	0.03	0.04	0.00	0.00	0.00	0.00
Superzyme-CC 2.5x (E2)^2^	0.00	0.00	0.02	0.02	0.00	0.00	0.02	0.02	0.00	0.00	0.02	0.02
*Calculated composition*												
Metabolizable energy, Kcal/kg	2900	2900	2900	2900	2950	2950	2950	2950	3050	3050	3050	3050
Crude protein, %	22.00	22.00	22.00	22.00	20.00	20.00	20.00	20.00	19.00	19.00	19.00	19.00
Calcium, %	0.96	0.96	0.96	0.96	0.80	0.80	0.80	0.80	0.74	0.74	0.74	0.74
Phosphorus, %	0.58	0.58	0.58	0.58	0.40	0.40	0.40	0.40	0.37	0.37	0.37	0.37
Sodium, %	0.18	0.18	0.18	0.18	0.18	0.18	0.18	0.18	0.18	0.18	0.18	0.18
Lysine, %	1.32	1.32	1.32	1.32	1.19	1.19	1.19	1.19	1.09	1.09	1.09	1.09
Methionine + Cystine, %	0.98	0.98	0.98	0.98	0.89	0.89	0.89	0.89	0.82	0.82	0.82	0.82

1 Supplied per kg of diet: 9,750 IU vitamin A, 2,000 IU vitamin D3, 50 IU vitamin E, 3.2 mg vitamin K, 8.6 mg riboflavin, 13.5 mg DL Ca-pantothenate, 0.023 mg vitamin B12, 40 mg niacin, 1.9 mg folic acid, 801 mg choline chloride, 0.25 mg biotin, 5.4 mg pyridoxine, 4 mg thiamine, 120 mg Manganous oxide, 110 mg Zinc oxide, 16 mg Copper sulfate.

2 Each gram of the enzyme product (Superzyme -CC 2.5x) provided: 2,875 XYL units of xylanase, 900 GLU units of glucanase, 542 INV units of invertase, 7,750 HUT units of Protease, 4,500 CMC units of cellulase, 18,125 FFA units of amylase and 60 GAL units of galactanase. Each kg of feed contains 575 XYL units of xylanase, 180 GLU units of glucanase, 108 INV units of invertase, 1,550 HUT units of Protease, 900 CMC units of cellulase, 3,625 FFA units of amylase and 12 GAL units of galactanase.

4 Starter, grower and finisher diets: Control, basal diet; 5PP, PP replacing 5% of the corn meal in the control diet; 5PP+E2, 5PP diet supplemented with E2; 10PP+E2: PP replacing 5% of the corn meal in the control diet and supplemented with E2.

***Measurements.*** Birds were weighed on d 8, 15, 25 and 33. Feed was added to the feeders as needed and the remaining feed was weighed on each scheduled weigh day and following any mortality event. When mortality occurred, feed consumption per bird within a pen was calculated by dividing the growth period into sub-periods based on the actual number of birds present. Average daily feed consumption was calculated as the total feed consumption per bird per period divided by the number of days in that period. Using these values, daily feed consumption, body weight gain and FCR per bird per period were calculated to assess growth performance. The flock performance was checked twice per day and mortality was recorded. On d 8 and d 33 of age, one bird per pen (random sex birds on d 8 and male birds on d 33) was randomly selected and euthanized for assessing gastrointestinal organ development. Intestinal segments (duodenum, jejunum, ileum and large intestine) and organs (yolk sac for d 8 only, crop, gizzard, liver, pancreas and ceca) were weighed and calculated as g/100 g of body weight. The length of the intestines was measured, and the weight-to-length ratio (mg/mm) was calculated.

### Statistical analysis

Data were analyzed using an ANOVA mixed model procedure in SAS v.9.4 (SAS Inc., Cary, NC, 2013). The statistical model for Study 1 included the fixed effects of potato peel meal inclusion level, enzyme supplementation and their interaction. For Study 2, each transportation basket was used as the experimental unit and transportation performance variables were analyzed using a one-way ANOVA with in-transit feeding practice as the fixed effect. For post-placement performance variables analysis, each floor pen was used as the experimental unit and variables were analyzed using a two-way ANOVA including the fixed effects of in-transit feeding practice, dietary treatment and their interaction. The assumptions of normality and homogeneity of variance of residuals were met before ANOVA analysis. In all cases, if significant effects were found, the Tukey-Kramer test was applied to compare means at the 5% significance level.

## Results and discussion

### Study 1: preliminary evaluation of PP in grower and finisher phases

Effects of PP inclusion and multi-carbohydrase supplementation on growth performance and digestive organ weight are presented in Table 4. Birds that were fed a 10PP diet showed a significantly lower body weight (*P* ≤ 0.05) on d 24 and 33 of age compared to those fed a diet with 0PP. During both grower and finisher phases, broilers fed the 10PP diet exhibited reduced body weight gain, lower feed consumption and consequently a poorer FCR (*P* ≤ 0.05), regardless of whether the diets were supplemented with enzyme (Table 4). There was no interaction effect (*P* > 0.05) between the inclusion level of PP and enzyme supplementation on growth performance and digestive organ weight (Table S1). The adverse effects on growth performance observed in the current study are likely attributed to the high fiber content in 10PP diets. Chemical analysis has shown that potato peels used in the current study contain 73% NDF, 59% ADF and overall high levels of NSP (Table 1). Based on these characteristics, it can be concluded that PP fiber is poorly soluble, highly lignified and rich in cellulose, as ADF accounts for 80% of NDF. This type of dietary fiber can be considered as a nutrient diluent, biologically inert, and when included at excessive levels, can impair nutrient availability and absorption in the small intestine. Our results align with some previous studies reporting that incorporating an agro byproduct with high fiber content to poultry feed reduces body weight as inclusion levels increase (Anaeto et al., 2009; Sklan et al., 2003). Walugembe et al. (2014) reported that high inclusion level of dietary fiber (6% and 8% of wheat bran from d 1-12 and d 13-21, respectively) suppressed daily body weight gain in broilers throughout the study. However, moderate dietary fiber inclusion has been shown to enhance growth performance. Broilers fed diets with a moderate level of dietary fiber (5% of pea hull) resulted in improved body weight gain and FCR from d 1 to 18 of age compared to those fed an excessive level (7.5%) of dietary fiber (Jiménez-Moreno et al., 2011). In the current study, the inclusion of 10% PP appears to exceed the beneficial threshold due to the high fiber content in potato peels.

**Table 4:** Effects of dietary potato peel treatments and enzyme supplementation on body weight (g bird^-1^), daily body weight gain (g bird^-1^ day^-1^), daily feed intake (g bird^-1^ day^-1^), feed conversion ratio and day 33 of age digestive organs weight (g/100g of body weight) of Cobb 500 broilers during the grower (d 15-35) and finisher (d 25-33) phases in Study 1.

Factors	Body weight		Daily body weight gain		Daily feed intake		Feed conversion ratio		Gizzard	Duodenum	Ileum	Large intestine
	D24	D33	D15-24	D25-33	D15-24	D25-33	D15-24	D25-33	D33	D33	D33	D33
*Potato Peel Meal (PP)*												
0PP	1755.2^a^	2866.3^a^	112.3^a^	123.5^a^	144.8^a^	209.7^a^	1.28^b^	1.70^b^	1.17^b^	0.84^b^	1.63^b^	0.12^b^
10PP	1472.0^b^	2483.2^b^	85.3^b^	112.4^b^	125.3^b^	200.4^b^	1.47^a^	1.79^a^	1.36^a^	1.05^a^	2.00^a^	0.16^a^
SEM	17.72	29.15	1.52	1.76	1.49	1.86	0.013	0.020	0.051	0.034	0.071	0.007
*Enzyme supplement (E)*												
No	1617.0	2680.3	98.9	118.2	135.5	205.5	1.37	1.75	1.22	0.96	1.89	0.15
Yes	1610.2	2669.2	98.7	117.7	134.5	204.6	1.37	1.74	1.31	0.92	1.74	0.13
SEM	17.72	29.15	1.52	1.76	1.49	1.86	0.013	0.020	0.051	0.034	0.071	0.007
*ANOVA P-value*												
PP	<0.01	<0.01	<0.01	<0.01	<0.01	<0.01	<0.01	<0.01	0.013	<0.01	<0.01	<0.01
E	0.790	0.791	0.935	0.842	0.665	0.735	0.965	0.907	0.231	0.419	0.141	0.124
PP x E	0.178	0.252	0.156	0.552	0.174	0.064	0.243	0.486	0.317	0.178	0.774	0.505

^a,b^ Means within a column with different letters differ significantly according to Tukey-Kramer test (α = 0.05).

Note: 0PP, basal diet; 10PP: PP replacing 10% of the corn meal in the basal diet.

Compared to birds fed 0PP, heavier (*P* ≤ 0.05) gizzard, duodenum, ileum, and large intestine were observed in the birds fed 10% PP diets, while the enzyme supplementation or its interaction with PP inclusion had no effect on digestive organ weight (*P* > 0.05) (Table 4). The increased gizzard weight observed in this study aligns with previous research, which reported heavier gizzards and small intestines in broilers fed high fiber diets (JøRgensen et al., 1996). The enlarged gizzard observed in the current study may reflect compensatory development in response to a high intake of dietary fiber from PP, which is rich in water-insoluble dietary fiber fractions such as lignin and cellulose, and consists of large particle size (4 mm) of PP. Sklan et al. (2003) reported that turkeys fed 60 g/kg of dietary fibre exhibited a longer duodenum and larger villus surface area than those fed 30 and 90 g/kg of dietary fiber. In the current study, heavier duodenum, ileum and large intestine were observed, but growth performance was suppressed, which may be attributed to increased digesta retention in the digestive tract rather than enhanced nutrient absorption. Abdel‐Hafeez et al. (2018) suggested that potato peels can be included in broiler diets at a level up to 15% when supplemented with multi-carbohydrase (Enziver®). In contrast to their findings, our results showed no effect of enzyme supplementation on growth performance or the weight of gastrointestinal organs. It can be attributed to a sixfold lower cellulase activity in the multi-carbohydrase, E1, used in Study 1, as well as to differences in the proportions of other enzyme activities between the two preparations. Although E1 still exhibits relatively high cellulase activity, it demonstrated limited capability to hydrolyze the polysaccharides present in PP. Given the suppressed growth performance observed in Study 1, a new enzyme blend (E2) with more target-specific activities towards PP was used in Study 2. E2 was formulated to provide higher cellulase, glucanase, and additional galactanase activities to improve NSP degradation, enhance nutrient utilization and mitigate the negative impacts of PP inclusion on growth. In addition, Study 2 evaluated graded PP inclusion levels, including the 10% PP used in Study 1 and an intermediate 5% level, to identify an optimal level that minimizes adverse effects on growth performance.

### Study 2: in-transit feeding and potato peel meal inclusion

***Body Weights, Cloaca Temperature and Chick Quality*.** No differences in body weight and cloaca temperature (*P* > 0.05) were found between the NFW and AFW groups before transport (Table 5). As expected, chicks with access to feed and water had less body weight loss or even gained weight during transportation compared to those without access. When body weight changes during transport were expressed as a percent of pre-transport body weight, chicks in the AFW group gained weight (2.47%), whereas those in the NFW group showed a decrease in body weight (-1.71%; *P* ≤ 0.05). This finding is consistent with the results reported by Boussaada et al. (2020), who found that the provision of feed and water during transportation resulted in less body weight loss than the no-fed group. The increased body weight after transportation found in the AFW group could also be attributed to the presence of feed in their gastrointestinal tract, as the crop fill score and digesta weights in the gizzard, jejunum and ileum in AFW chicks were significantly higher (*P* ≤ 0.05) than those in the NFW group (Table 5). No differences in yolk-free body weight, the relative weight of liver, emptied gizzard, cecum and intestines (*P* > 0.05) were found between NFW and AFW (Table 5).

**Table 5:** Effect of access to feed and water during a 4-h transportation on body weight, cloaca temperature, plasma glucose and corticosterone level, and gastrointestinal organ weight of Ross 308 broilers post transportation in Study 2.

Parameter	In-transit feeding^1^		SEM	*P*-value
	NFW	AFW		
*Chick quality*				
Body weight prior transport (g bird^-^1)	37.8	37.8	0.18	0.939
Body weight post transport (g bird^-^1)	37.2^b^	38.8^a^	0.27	<0.01
Body weight gain (%)	-1.71^b^	2.47^a^	0.401	<0.01
Cloaca temperature prior transport (°C)	38.7	38.6	0.07	0.651
Cloaca temperature post transport (°C)	38.8^b^	39.3^a^	0.07	<0.01
Glucose level post transport (mmol/L)	13.4	12.6	0.32	0.138
Corticosterone level post transport (ng/mL)	15.12^a^	8.17^b^	1.482	<0.01
Crop fill score^2^	0.0^b^	2.8^a^	0.18	<0.01
Feed consumption (g bird^-^1)	0.0	1.3	0.05	<0.01
Water consumption (g bird^-^1)	0.0	2.5	0.15	<0.01
Yolk-free body weight (g bird^-^1)	32.5	34.4	0.79	0.122
*Gastrointestinal organ weight*				
Relative yolk residual weight (%)	12.33	10.42	0.688	0.078
Relative gizzard content weight (%)	0^b^	2.05^a^	0.131	<0.01
Relative gizzard weight (%)	5.78	5.39	0.152	0.105
Relative liver weight (%)	3.39	3.37	0.087	0.897
Relative duodenum content weight (%)	0.48	0.49	0.038	0.886
Relative empty duodenum weight (%)	0.42	0.46	0.028	0.305
Relative jejunum content weight (%)	0.77^b^	1.11^a^	0.080	<0.05
Relative empty jejunum weight (%)	0.56	0.63	0.036	0.169
Relative ileum content weight (%)	0.58^b^	0.83^a^	0.044	<0.01
Relative empty ileum weight (%)	0.49	0.54	0.035	0.336
Relative cecal content weight (%)	0.41	0.32	0.048	0.196
Relative empty cecum weight (%)	0.32	0.32	0.025	0.959

^a,b^ Means within a row with different letters differ significantly according to Tukey-Kramer test (α = 0.05).

1 In-transit feeding: NFW: No access to feed and water during transportation; AFW: Access to feed and water during transportation.

2 Crop fill score: Score 0-empty; score 1-feed only; score 2-water only; score 3-feed and water, full, soft and rounded.

During the 4-h of transportation, the feed-to-water consumption ratio in the AFW group was approximately 1:1.9 (Table 5, 1.3±0.05 g of feed/chick and 2.5±0.15 g of water/chick, respectively), which indicates the importance of water availability to support efficient feed digestion. A similar feed-water consumption ratio was reported by Pesti et al. (1985), together with crop fill score, indicating that the current setup enables chicks to utilize both feeder and water drinkers efficiently. Water is an essential nutrient that receives little attention in early feeding practice. Having access to fresh water is particularly important for compensating for any dehydration that may result from the hatching process and post-hatch handling. However, due to the design of transportation baskets, the provision of fresh water is often impractical. In the present study, we modified the transportation baskets by attaching water drinkers through a simple adaptation of existing equipment. The approach offers a practical solution for producers who are aiming to implement the practice of providing water during transportation at a minimum cost.

The in-transit feeding practice did not affect blood glucose level post-transportation (*P* > 0.05). AFW chicks exhibited improved resilience to transport stress with a higher cloaca temperature (*P* ≤ 0.05) and lower corticosterone level post-transportation (*P* ≤ 0.05) as compared with the NFW chicks (Table 5). The optimal cloaca temperature for day-old chicks ranges from 39.4°C to 40.5°C (Aviagen, 2025). In the present study, the cloaca temperature before and post transport was 38.7°C and 38.8°C for NFW chicks, suggesting that chicks experienced mild cold stress under the current transport conditions (25°C and 33% relative humidity). To our knowledge, this is the first study to investigate the impact of early feeding during transportation on the thermoregulation of young chicks. Our results demonstrated that the provision of feed and water during transportation not only mitigates the transport-related stress, but also supports metabolic heat production, potentially helping chicks to maintain the optimal body temperature under handling and suboptimal transportation conditions. Hollemans et al. (2018) reported that newly hatched chicks having access to feed and water in an on-farm hatching system had a higher cloaca temperature at placement than those without access. The same study also investigated the impact of early nutrient access and transportation on fear response of broilers. Chicks in the early nutrient access group expressed higher fear responses with a longer latency to stand up during the tonic immobility test on d 3 of age than those without access to feed and water before placement. The authors speculated that early nutrient access may not only accelerate muscle and organ development in chicks but also stimulate the neural development and enhance cognitive ability of young broilers. In the current study, the lower plasma corticosterone level observed in the AFW group provides direct evidence to support the importance of providing feed and water during transportation to mitigate transport stress and improved welfare of day-old chicks.

***Body Weight, Feed Consumption and FCR During the Grow-out Phase.*** Results of body weight, feed consumption and FCR are presented in Tables 6, Table 7, Table 8, respectively. AFW resulted in heavier body weight (*P* ≤ 0.05) than NFW chicks until d 8 of age (Table 6). The improved body weight at an early age through early feeding practice agrees with previous findings (Liu et al., 2020; Uni et al., 2003). Nutrient utilization is largely affected by the development and maturation of the gastrointestinal tract. Early access to carbohydrate-based feed could stimulate the gastrointestinal tract development and digestive enzyme secretion, which enables more efficient nutrient digestion and absorption. Previous studies have demonstrated that delayed feed access not only disrupts the physiological development of newly hatched chicks but also suppresses growth in the long term (Liu et al., 2020; Uni et al., 2003; Özlü et al., 2022). However, in-transit feeding practice did not affect subsequent body weight (*P* > 0.05) (Table 6). The in-transit feeding practice with an application duration of 4 hours supports the early growth of broilers in the current study. The beneficial effects of in-transit feeding may become more pronounced in scenarios where newly hatched chicks need to experience a longer duration of transportation. The adverse effect of delayed access to feed on gut morphology and function has been reported by Liu et al. (2020), who found that increased villus surface area and goblet cell proliferation in the duodenum when chicks had immediate access to feed and water after hatch. Additionally, delayed access to feed and water downregulated the *Muc2* expression, which may compromise gut health and subsequently impair nutrition absorption and feed efficiency. Considering the variation in hatching time and the duration of the hatchery process, chicks can experience up to 72 h of feed and water deprivation. Therefore, access to feed and water during the period between hatch and arrival at the grow-out facility could be critical to support the physiological development of chicks. Dietary treatment or its interaction with in-transit feeding practice did not affect body weight (*P* > 0.05) throughout the study (d 0-33), indicating that incorporating PP up to 10% and supplemented with E2 did not compromise broiler growth.

**Table 6:** Effects of in-transit feeding and dietary potato peel meal (PP) on body weight (g bird^-^1) in Ross 308 broilers in Study 2.

Treatment	Body weight (g bird^-1^)				
	0 d	8 d	15 d	25 d	33 d
*In-transit feeding*^1^					
NFW	37.1^b^	141.3^b^	392.2	1225.7	2147.1
AFW	38.7^a^	149.7^a^	404.6	1231.1	2169.5
SEM	0.16	2.68	9.92	23.83	35.62
*Dietary treatment*^2^					
Control	37.6	146.8	403.1	1237.8	2151.9
5PP	38.0	145.5	386.7	1215.7	2137.6
5PP+E2	37.9	152.0	421.8	1271.9	2227.5
10PP+E2	38.1	137.8	382.1	1188.1	2116.2
SEM	0.22	3.79	14.02	33.70	50.38
*ANOVA P-value*					
In-transit feeding	<0.01	<0.05	0.388	0.874	0.662
Dietary treatment	0.472	0.106	0.218	0.376	0.452
In-transit feeding x Dietary treatment	0.125	0.152	0.297	0.379	0.731

^a,b^ Means within a column with different letters differ significantly according to Tukey-Kramer test (α = 0.05).

1 In-transit feeding: NFW: No access to feed and water during transportation; AFW: Access to feed and water during transportation.

2 Dietary treatment: Control, basal diet; 5PP, PP replacing 5% of the corn meal in the control diet; 5PP+E2, 5PP diet supplemented with E2; 10PP+E2: PP replacing 5% of the corn meal in the control diet and supplemented with E2.

**Table 7:** Effects of in-transit feeding and dietary potato peel (PP) meal on feed consumption (g bird^-^1 day^-^1) in Ross 308 broilers in Study 2.

Treatment	Feed consumption (g bird^-1^ day^-1^)			
	0-8 d	9-15 d	16-25 d	26-33 d
*In-transit feeding*^1^				
NFW	20.5	48.4	110.8	174.0
AFW	21.2	47.5	111.2	174.1
SEM	0.34	0.78	2.10	2.67
*Dietary treatment*^2^				
Control	20.5	49.1^ab^	109.9	172.1
5PP	20.2	45.9^b^	110.7	172.8
5PP+E2	20.8	50.3^a^	114.7	175.1
10PP+E2	21.8	46.5^ab^	108.8	176.3
SEM	0.49	1.10	2.97	3.78
*ANOVA P-value*				
In-transit feeding	0.174	0.461	0.884	0.978
Dietary treatment	0.129	<0.05	0.538	0.844
In-transit feeding x Dietary treatment	0.615	0.212	0.415	0.843

^a,b^Means within a column with different letters differ significantly according to Tukey-Kramer test (α = 0.05).

1 In-transit feeding: NFW: No access to feed and water during transportation; AFW: Access to feed and water during transportation.

2 Dietary treatment: Control, basal diet; 5PP, PP replacing 5% of the corn meal in the control diet; 5PP+E2, 5PP diet supplemented with E2; 10PP+E2: PP replacing 5% of the corn meal in the control diet and supplemented with E2.

**Table 8:** Effects of in-transit feeding and dietary potato peel (PP) meal on feed conversion ratio in Ross 308 broilers in Study 2.

Treatment	Feed conversion ratio			
	0-8 d	9-15 d	16-25 d	26-33 d
*In-transit feeding*^1^				
NFW	1.58	1.35	1.33	1.51
AFW	1.55	1.32	1.35	1.49
SEM	0.047	0.032	0.008	0.011
*Dietary treatment*^2^				
Control	1.51^ab^	1.34	1.32	1.51
5PP	1.52^ab^	1.34	1.34	1.50
5PP+E2	1.47^b^	1.31	1.35	1.47
10PP+E2	1.76^a^	1.35	1.35	1.52
SEM	0.066	0.045	0.011	0.015
*ANOVA P-value*				
In-transit feeding	0.569	0.468	0.176	0.113
Dietary treatment	<0.05	0.925	0.154	0.120
In-transit feeding x Dietary treatment	0.085	0.695	0.672	<0.05

^a,b^Means within a column with different letters differ significantly according to Tukey-Kramer test (α = 0.05).

1 In-transit feeding: NFW: No access to feed and water during transportation; AFW: Access to feed and water during transportation.

2 Dietary treatment: Control, basal diet; 5PP, PP replacing 5% of the corn meal in the control diet; 5PP+E2, 5PP diet supplemented with E2; 10PP+E2: PP replacing 5% of the corn meal in the control diet and supplemented with E2.

The average daily body weight gain was not affected (*P* > 0.05) by the main effect, in-transit feeding practice, dietary treatment, or their interaction (Table S2). The supplementation of E2 mitigated the negative effect on body weight gain observed in Study 1 when birds fed the same potato peel inclusion level (10%) but supplemented with E1. Birds fed PP diets having similar body weight and body weight gain to those fed control diet suggest that reducing PP inclusion level to 5% or supplementing a suitable enzyme blend can enable effective digestion of PP without compromising growth performance. Understanding the composition and properties of dietary fiber specific to given ingredients is essential for the strategic selection of exogenous carbohydrates. Given the structural complexity and diversity of dietary fiber, analyzing the fiber fractions, particularly the NSP profile, offers valuable information on the specificity of the substrate. The effectiveness of enzyme supplementation depends on the specificity for the substrate, and the substrate’s accessibility and abundance (Aftab and Bedford, 2018). Therefore, to maximize nutrient release from fiber-rich feed ingredients, enzyme blends must be carefully formulated by addressing the specificity of their NSP profile to aim for efficient fiber degradation and improve feed efficiency. Supplementation of the PP diet with E2 helped counteract the negative impact of PP dietary fiber on growth performance when PP was included at up to 10% in Study 2. E2 supplementation also alleviated the body weight reduction observed in Study 1 when 10% PP was supplemented with E1. Qualitative and quantitative analyses of NSP in intestinal digesta, as well as profiling of short-chain fatty acids in cecal content, are warranted in future studies to provide more comprehensive insight into the efficacy of exogenous enzymes in enhancing fiber digestion.

**Fig. 2:**
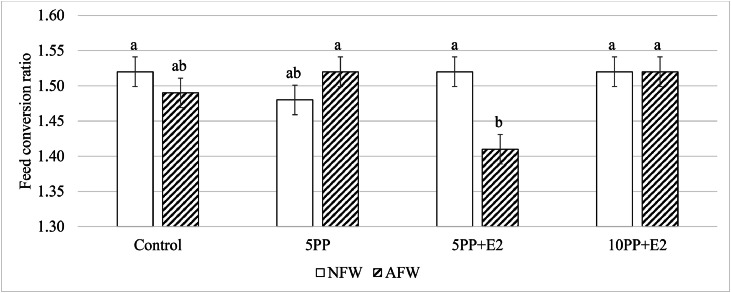
Effects of in-transit feeding and dietary potato peel meal (PP) on feed conversion ratio for the finisher phase (day 26-33 of age) in Ross 308 broilers in Study 2.

^a,b^Means lacing a common letter differ significantly according to Tukey-Kramer test (α = 0.05). Note: NFW: No access to feed and water during transportation; AFW: Access to feed and water during transportation; Control, basal diet; 5PP, PP replacing 5% of the corn meal in the control diet; 5PP+E2, 5PP diet supplemented with E2; 10PP+E2: PP replacing 5% of the corn meal in the control diet and supplemented with E2.

Feed consumption was not affected (*P* > 0.05) by the in-transit feeding practice throughout the study (Table 7). However, higher feed consumption (*P* ≤ 0.05) was observed in the chicks fed 5PP+E2 than those fed 5PP from d 9-15 of age. The increased feed consumption by enzyme supplement in later age agrees with the study of Singh et al. (2017) where broiler diets supplemented with multi-enzymes did not affect feed consumption for the first two weeks, while a higher feed consumption was found from d 15-21 of age when fed low fiber content diets. Compared to 10PP+E2, FCR was improved (*P* ≤ 0.05) in birds fed 5PP+E2 diet during the first week (Table 8). Young broilers may not be able to produce sufficient enzymes to effectively digest the diet containing 10% PP, as previous research has shown that the activity of pancreatic enzymes is the lowest at hatch and increases with age in broilers (Mahagna et al., 1995). FCR during the finisher phase was affected by a two-way interaction between in-transit feeding practice and dietary treatment (*P* ≤ 0.05) and the corresponding data are presented in Fig. 2. No difference in FCR (*P* > 0.05) was observed among dietary treatments in the NFW group (Fig. 2). However, birds fed 5PP+E2 had an improved FCR (*P* ≤ 0.05) in the finisher phase, compared to those fed 5PP and 10PP+E2 when chicks having access to feed and water during transportation. In addition, the in-transit feeding practice improved FCR (*P* ≤ 0.05) during d 26-33 when fed the same diet (5PP+E2). These results demonstrated that early access to feed and water can provide a long-term effect on aiding the digestion of diets with a moderate level of fiber inclusion. The benefits of early feeding strategies on birds’ development later in life have been reported in previous studies. Improved body weight gain and longer duodenum length were found in birds having early access to feed and water (Boussaada et al., 2020; Deines et al., 2021). Accelerated development of the gastrointestinal tract allows more efficient nutrient digestion and absorption. Broilers fed 10PP+E2 had a poorer FCR than those fed 5PP+E2, suggesting that the optimal level of exogenous enzyme inclusion might depend on the proportion of high-fiber ingredients in the feed.

***Organ Weight, Intestinal Weight and Length, and Mortality.*** The results of gastrointestinal tract organ weight on d 8 and 33 are shown in the supplementary tables (d 8: Table S3 & S4, d 33: Table S5 & S6). Crop, gizzard, liver, pancreas and cecum weights, as a percentage of body weight were not affected (*P* > 0.05) by the in-transit feeding practice, dietary treatment or their interaction effect on d 8 or 33 of age. The main effects and their interaction did not significantly affect (*P* > 0.05) the relative weight of duodenum, jejunum and ileum, or their weight-to-length ratio. These findings suggest that in-transit feeding practice or dietary treatment did not influence gastrointestinal tract development under the given experimental conditions.

Mortality rate during the first 8 d was affected by in-transit feeding (Table 9). Chicks in the NFW group had a higher mortality rate (*P* ≤ 0.05) than those in AFW. There was no effect (*P* > 0.05) of dietary treatment or its interaction with in-transit feeding practice on cumulative mortality during the entire production phase (Table 9). Increased mortality related to transport stress has been reported in both newly hatched chicks and market-gate broilers (Chauvin et al., 2011; Vieira et al., 2019). Xu et al. (2022) found that decreased total antioxidant capacity content and increased catalase activity were observed in heart tissue after 4 h of transportation. The reduced level of total antioxidant capacity indicates that newly hatched chicks experienced oxidative stress and could potentially impair immune function, reduce disease resistance, and even lead to mortality. However, no difference in mortality was observed among chicks transported for 0, 4 and 10 h when under 23 to 29°C and 34-45% RH (Bergoug et al., 2013). It is important to note that many studies evaluating the impact of delayed feed access on bird development and health were conducted in the environmentally controlled grow-out facility (Gaweł et al., 2022; Kang et al., 2018; Wijnen et al., 2022). The stress stimulus from the delayed feeding or simulated transportation condition may not fully capture the stressors present during the actual transportation process, such as vibration and intermittent stops between farms. In contrast, the current study simulated practical commercial transportation conditions. Lower plasma corticosterone level following transportation and reduced mortality rate during the first week suggest that in-transit feeding practice can mitigate transport-related stress, enhance the welfare of day-old chicks and support subsequent growth and health of broilers.

**Table 9:** Effects of in-transit feeding and dietary potato peel (PP) meal on mortality rate in Ross 308 broilers in Study 2.

Treatment	Mortality rate (%)	
	0-8 d	0-33 d
*In-transit feeding*^1^		
NFW	1.74^a^	2.78
AFW	0.00^b^	1.41
SEM	0.491	0.741
*Dietary treatment*^2^		
Control	1.39	2.08
5PP	0.70	2.09
5PP+E2	0.70	2.09
10PP+E2	0.70	2.12
SEM	0.695	1.047
*ANOVA P-value*		
In-transit feeding	<0.05	0.208
Dietary treatment	0.860	1.000
In-transit feeding x Dietary treatment	0.860	0.351

^a,b^Means within a column with different letters differ significantly according to Tukey-Kramer test (α = 0.05).

1 In-transit feeding: NFW: No access to feed and water during transportation; AFW: Access to feed and water during transportation.

2 Dietary treatment: Control, basal diet; 5PP, PP replacing 5% of the corn meal in the control diet; 5PP+E2, 5PP diet supplemented with E2; 10PP+E2: PP replacing 5% of the corn meal in the control diet and supplemented with E2.

In conclusion, dietary supplementation with a suitable multi-carbohydrase (E2) enabled up to 10% PP inclusion without compromising growth performance. In-transit feeding practice not only helped chicks maintain body temperature homeostasis and mitigate transport-induced stress but also improved FCR when a moderate level (5%) of PP was included during the finisher phase. These findings provide poultry producers with practical feeding strategies that are more sustainable and cost-effective.

## CRediT authorship contribution statement

**Xujie Li:** Writing – review & editing, Writing – original draft, Visualization, Validation, Software, Resources, Project administration, Methodology, Investigation, Funding acquisition, Formal analysis, Data curation, Conceptualization. **Jing Lu:** Writing – review & editing, Methodology, Data curation. **Bruce Rathgeber:** Writing – review & editing, Supervision, Methodology, Funding acquisition. **Anna Rogiewicz:** Writing – review & editing, Validation, Methodology, Formal analysis, Data curation. **Stephanie Collins:** Writing – review & editing, Supervision, Project administration, Methodology, Funding acquisition, Data curation, Conceptualization.

## Disclosures

The authors declare the following financial interests/personal relationships which may be considered as potential competing interests: Xujie Li reports financial support was provided by The McCain Foundation. Xujie Li reports equipment, drugs, or supplies was provided by CBS Bio Platforms Inc. If there are other authors, they declare that they have no known competing financial interests or personal relationships that could have appeared to influence the work reported in this paper.

## Appendix Supplementary materials

Image, application 1

## Footnotes

Supplementary material associated with this article can be found, in the online version, at doi:10.1016/j.psj.2026.106809.
