# Supplementary material for: Does in-transit feeding of day-old chicks enhance potato peel meal utilization? Impact on transport stress, gastrointestinal organ weight and performance in broilers
Source: Poult Sci. 2026 Mar 17;105(6):106809. doi: 10.1016/j.psj.2026.106809 (PMC13052023; doi:10.1016/j.psj.2026.106809)
Supplement: Supplementary file 1 [file mmc1.docx]

Table S1: Interaction effects of dietary potato peel treatments and enzyme supplementation during the grower (d 15-35) and finisher (d 25-33) phases on body weight (g bird^-1^), daily body weight gain (g bird^-1^ day^-1^), daily feed intake (g bird^-1^ day^-1^), feed conversion ratio and day 33 of age gastrointestinal organs weight (g/100g of body weight) of Cobb 500 broilers in Study 1.

^1^D14: Average body weight of birds at 14 days of age, immediately before the dietary treatments containing potato peel meal were applied.

Note: 0PP, basal diet; 0PP+E1, basal diet supplemented with E1; 10PP: PP replacing 10% of the corn meal in the basal diet; 10PP+E1: 10PP diet supplemented with E1.

| Factors | Body weight | | | Daily body weight gain | | Daily feed intake | | Feed conversion ratio | | Gizzard | Duodenum | Ileum | Large intestine |
| --- | --- | --- | --- | --- | --- | --- | --- | --- | --- | --- | --- | --- | --- |
|  | D14^1^ | D24 | D33 | D15-24 | D25-33 | D15-24 | D25-33 | D15-24 | D25-33 | D33 | D33 | D33 | D33 |
| 0PP | 633.9 | 1776.1 | 2896.2 | 114.0 | 124.5 | 146.7 | 212.8 | 1.27 | 1.71 | 1.08 | 0.83 | 1.69 | 0.13 |
| 0PP+E1 | 625.5 | 1734.3 | 2836.4 | 110.7 | 122.5 | 142.8 | 206.7 | 1.29 | 1.69 | 1.25 | 0.86 | 1.57 | 0.11 |
| 10PP | 626.4 | 1457.9 | 2464.4 | 83.8 | 111.9 | 124.2 | 198.2 | 1.48 | 1.78 | 1.35 | 1.10 | 2.09 | 0.16 |
| 10PP+E1 | 621.0 | 1486.2 | 2502.1 | 86.8 | 112.9 | 126.3 | 202.5 | 1.46 | 1.80 | 1.37 | 0.99 | 1.91 | 0.15 |
| SEM | 3.59 | 25.05 | 41.21 | 2.15 | 2.43 | 2.09 | 2.62 | 0.018 | 0.028 | 0.072 | 0.048 | 0.101 | 0.010 |
| *ANOVA P-value* | |  |  |  |  |  |  |  |  |  |  |  |  |
| PP | 0.115 | <0.01 | <0.01 | <0.01 | <0.01 | <0.01 | <0.01 | <0.01 | <0.01 | 0.013 | <0.01 | <0.01 | <0.01 |
| E | 0.074 | 0.790 | 0.791 | 0.935 | 0.842 | 0.665 | 0.735 | 0.965 | 0.907 | 0.231 | 0.419 | 0.141 | 0.124 |
| PP x E | 0.684 | 0.178 | 0.252 | 0.156 | 0.552 | 0.174 | 0.064 | 0.243 | 0.486 | 0.317 | 0.178 | 0.774 | 0.505 |

Table S2. Effects of in-transit feeding and dietary potato peel (PP) meal on body weight gain (g bird^-1^ day^-1^) in Ross 308 broilers in Study 2.

| Treatment | Body weight gain (g bird^-1^ day^-1^) | | | |
| --- | --- | --- | --- | --- |
|  | 0-8 d | 9-15 d | 16-25 d | 26-33 d |
| *In-transit feeding^1^* | | | | |
| NFW | 13.0 | 35.8 | 83.3 | 115.2 |
| AFW | 13.9 | 36.4 | 82.7 | 117.3 |
| SEM | 0.32 | 1.19 | 1.46 | 1.67 |
| *Dietary treatment^2^* | | | | |
| Control | 13.7 | 36.6 | 83.5 | 114.3 |
| 5PP | 13.4 | 34.5 | 82.9 | 115.2 |
| 5PP+E2 | 14.3 | 38.5 | 85.0 | 119.5 |
| 10PP+E2 | 12.5 | 34.9 | 80.6 | 116.0 |
| SEM | 0.46 | 1.69 | 2.07 | 2.36 |
| *ANOVA P-value* |  |  |  |  |
| In-transit feeding | 0.075 | 0.734 | 0.743 | 0.384 |
| Dietary treatment | 0.077 | 0.335 | 0.521 | 0.456 |
| In-transit feeding x Dietary treatment | 0.111 | 0.447 | 0.465 | 0.558 |

^1^In-transit feeding: NFW: No access to feed and water during transportation; AFW: Access to feed and water during transportation.

^2^Dietary treatment: Control, basal diet; 5PP, PP replacing 5% of the corn meal in the control diet; 5PP+E2, 5PP diet supplemented with E2; 10PP+E2: PP replacing 5% of the corn meal in the control diet and supplemented with E2.

Table S3. Effect of in-transit feeding and dietary potato peel (PP) meal on glucose and organ weight of Ross 308 broilers at day 8 of age in Study 2.

| Treatment | Organ development | | | | | | |
| --- | --- | --- | --- | --- | --- | --- | --- |
|  | Glucose level (mmol/L) | Relative crop weight (%) | Relative gizzard weight (%) | Relative yolk sac weight (%) | Relative liver weight (%) | Relative Pancreas weight (%) | Relative cecum weight (%) |
| *In-transit feeding^1^* | | | | | |  |  |
| NFW | 12.2 | 0.58 | 3.93 | 0.07 | 3.86 | 0.48 | 1.14 |
| AFW | 11.8 | 0.58 | 4.04 | 0.10 | 3.98 | 0.52 | 1.09 |
| SEM | 0.32 | 0.026 | 0.217 | 0.044 | 0.159 | 0.024 | 0.086 |
| *Dietary treatment^2^* | | | | | |  |  |
| Control | 11.7 | 0.56 | 4.09 | 0.08 | 3.70 | 0.52 | 1.20 |
| 5PP | 12.7 | 0.55 | 3.73 | 0.09 | 3.89 | 0.51 | 1.05 |
| 5PP+E2 | 11.7 | 0.59 | 3.65 | 0.09 | 3.82 | 0.51 | 0.93 |
| 10PP+E2 | 12.0 | 0.63 | 4.45 | 0.09 | 4.27 | 0.47 | 1.28 |
| SEM | 0.45 | 0.037 | 0.307 | 0.063 | 0.225 | 0.033 | 0.121 |
| *ANOVA P-value* |  |  |  |  |  |  |  |
| In-transit feeding | 0.424 | 0.899 | 0.726 | 0.607 | 0.604 | 0.260 | 0.670 |
| Dietary treatment | 0.409 | 0.462 | 0.271 | 0.999 | 0.353 | 0.620 | 0.224 |
| In-transit feeding x Dietary treatment | 0.602 | 0.819 | 0.944 | 0.205 | 0.253 | 0.561 | 0.947 |

^1^In-transit feeding: NFW: No access to feed and water during transportation; AFW: Access to feed and water during transportation.

^2^Dietary treatment: Control, basal diet; 5PP, PP replacing 5% of the corn meal in the control diet; 5PP+E2, 5PP diet supplemented with E2; 10PP+E2: PP replacing 5% of the corn meal in the control diet and supplemented with E2.

Table S4. Effect of in-transit feeding and dietary potato peel (PP) meal on small intestine and large intestine development of Ross 308 broilers at day 8 of age in Study 2.

^1^In-transit feeding: NFW: No access to feed and water during transportation; AFW: Access to feed and water during transportation.

^2^Dietary treatment: Control, basal diet; 5PP, PP replacing 5% of the corn meal in the control diet; 5PP+E2, 5PP diet supplemented with E2; 10PP+E2: PP replacing 5% of the corn meal in the control diet and supplemented with E2.

| Treatment | Organ development | | | | | | | |
| --- | --- | --- | --- | --- | --- | --- | --- | --- |
|  | Relative duodenum weight (%) | Duodenum weight to length ratio (mg/mm) | Relative jejunum weight (%) | Jejunum weight to length ratio (mg/mm) | Relative ileum weight (%) | Ileum weight to length ratio (mg/mm) | Relative large intestine weight (%) | Large intestine weight to length ratio (mg/mm) |
| *In-transit feeding^1^* | | | | | |  |  |  |
| NFW | 2.01 | 18.55 | 2.73 | 11.20 | 1.92 | 7.43 | 0.28 | 11.38 |
| AFW | 2.11 | 18.66 | 2.63 | 1.56 | 1.83 | 7.37 | 0.28 | 11.23 |
| SEM | 0.081 | 0.913 | 0.070 | 0.7033 | 0.062 | 0.569 | 0.017 | 0.616 |
| *Dietary treatment^2^* | | | | | |  |  |  |
| Control | 2.03 | 19.04 | 2.50 | 11.48 | 1.79 | 7.43 | 0.27 | 10.92 |
| 5PP | 2.02 | 18.26 | 2.77 | 11.03 | 1.80 | 7.01 | 0.28 | 10.48 |
| 5PP+E2 | 2.08 | 18.97 | 2.73 | 11.85 | 1.92 | 7.95 | 0.30 | 12.80 |
| 10PP+E2 | 2.11 | 18.15 | 2.73 | 11.17 | 1.98 | 7.21 | 0.27 | 11.01 |
| SEM | 0.114 | 1.291 | 0.099 | 0.965 | 0.088 | 0.805 | 0.024 | 0.871 |
| *ANOVA P-value* |  |  |  |  |  |  |  |  |
| In-transit feeding | 0.379 | 0.912 | 0.325 | 0.723 | 0.322 | 0.947 | 0.907 | 0.870 |
| Dietary treatment | 0.916 | 0.941 | 0.260 | 0.936 | 0.381 | 0.858 | 0.738 | 0.284 |
| In-transit feeding x Dietary treatment | 0.868 | 0.860 | 0.120 | 0.936 | 0.805 | 0.931 | 0.865 | 0.243 |

Table S5. Effect of in-transit feeding and dietary potato peel (PP) meal on organ weight of Ross 308 broilers at day 33 of age in Study 2.

| Treatment | Organ development | | | | |
| --- | --- | --- | --- | --- | --- |
|  | Relative crop weight (%) | Relative gizzard weight (%) | Relative liver weight (%) | Relative Pancreas weight (%) | Relative cecum weight (%) |
| *In-transit feeding^1^* | | | | | |
| NFW | 0.28 | 1.15 | 1.99 | 0.21 | 0.52 |
| AFW | 0.27 | 1.15 | 1.98 | 0.21 | 0.54 |
| SEM | 0.011 | 0.048 | 0.047 | 0.008 | 0.042 |
| *Dietary treatment^2^* | | | | | |
| Control | 0.27 | 1.14 | 2.04 | 0.22 | 0.55 |
| 5PP | 0.27 | 1.12 | 2.03 | 0.21 | 0.56 |
| 5PP+E2 | 0.29 | 1.12 | 1.88 | 0.20 | 0.52 |
| 10PP+E2 | 0.26 | 1.21 | 1.99 | 0.23 | 0.48 |
| SEM | 0.015 | 0.068 | 0.067 | 0.011 | 0.060 |
| *ANOVA P-value* |  |  |  |  |  |
| In-transit feeding | 0.672 | 0.993 | 0.828 | 0.892 | 0.838 |
| Dietary treatment | 0.723 | 0.735 | 0.358 | 0.487 | 0.807 |
| In-transit feeding x Dietary treatment | 0.497 | 0.877 | 0.097 | 0.214 | 0.924 |

^1^In-transit feeding: NFW: No access to feed and water during transportation; AFW: Access to feed and water during transportation.

^2^Dietary treatment: Control, basal diet; 5PP, PP replacing 5% of the corn meal in the control diet; 5PP+E2, 5PP diet supplemented with E2; 10PP+E2: PP replacing 5% of the corn meal in the control diet and supplemented with E2.

Table S6. Effect of in-transit feeding and dietary potato peel (PP) meal on small intestine and large intestine development of Ross 308 broilers at day 33 of age in Study 2.

| Treatment | Organ development | | | | | | | |
| --- | --- | --- | --- | --- | --- | --- | --- | --- |
|  | Relative duodenum weight (%) | Duodenum weight to length ratio (mg/mm) | Relative jejunum weight (%) | Jejunum weight to length ratio (mg/mm) | Relative ileum weight (%) | Ileum weight to length ratio (mg/mm) | Relative large intestine weight (%) | Large intestine weight to length ratio (mg/mm) |
| *In-transit feeding^1^* | | | | | |  |  |  |
| NFW | 0.51 | 41.16 | 1.04 | 37.10 | 0.87 | 30.51 | 0.12 | 45.70 |
| AFW | 0.53 | 42.26 | 1.02 | 38.53 | 0.90 | 21.11 | 0.12 | 43.71 |
| SEM | 0.020 | 1.981 | 0.030 | 1.209 | 0.026 | 1.373 | 0.005 | 2.169 |
| *Dietary treatment^2^* | | | | | |  |  |  |
| Control | 0.53 | 41.50 | 1.07 | 37.41 | 0.89 | 29.71 | 0.12 | 45.04 |
| 5PP | 0.51 | 43.41 | 1.05 | 37.68 | 0.91 | 32.08 | 0.12 | 45.57 |
| 5PP+E2 | 0.48 | 39.69 | 0.96 | 38.13 | 0.82 | 31.48 | 0.12 | 44.15 |
| 10PP+E2 | 0.56 | 42.24 | 1.04 | 38.03 | 0.92 | 31.97 | 0.12 | 44.07 |
| SEM | 0.028 | 1.795 | 0.043 | 1.710 | 0.036 | 1.941 | 0.007 | 3.067 |
| *ANOVA P-value* |  |  |  |  |  |  |  |  |
| In-transit feeding | 0.641 | 0.564 | 0.745 | 0.417 | 0.396 | 0.423 | 0.595 | 0.525 |
| Dietary treatment | 0.337 | 0.537 | 0.278 | 0.990 | 0.276 | 0.812 | 0.842 | 0.982 |
| In-transit feeding x Dietary treatment | 0.501 | 0.724 | 0.674 | 0.335 | 0.529 | 0.507 | 0.252 | 0.564 |

^1^In-transit feeding: NFW: No access to feed and water during transportation; AFW: Access to feed and water during transportation.

^2^Dietary treatment: Control, basal diet; 5PP, PP replacing 5% of the corn meal in the control diet; 5PP+E2, 5PP diet supplemented with E2; 10PP+E2: PP replacing 5% of the corn meal in the control diet and supplemented with E2.
